# Crystallization Kinetics in BaTiO_3_ Synthesis from Hydrate Precursors via Microwave-Assisted Heat Treatment

**DOI:** 10.3390/nano11030754

**Published:** 2021-03-17

**Authors:** Han-Sol Yun, Byeong-Gyu Yun, So-Young Shin, Dae-Yong Jeong, Nam-Hee Cho

**Affiliations:** Department of Materials Science and Engineering, Inha University, Nam-gu, Incheon 22212, Korea; hsyun4920@naver.com (H.-S.Y.); ybg326@naver.com (B.-G.Y.); 22201267@inha.edu (S.-Y.S.); dyjeong@inha.ac.kr (D.-Y.J.)

**Keywords:** microwave-assisted heating, hydrate precursors, charged radicals, crystallization kinetics, real-time measurements

## Abstract

The crystallization kinetics in BaTiO_3_ synthesis from hydrate precursors via microwave-assisted heating (MWH) were investigated. The structural and chemical features of powders synthesized via MWH and conventional heating (CH) were compared. The charged radicals generated under microwave irradiation were identified by chemical analysis and real-time charge flux measurements. Using Ba(OH)_2_∙H_2_O (BH1), Ba(OH)_2_ (BH0), and BaCO_3_ (BC) as the precursors for a Ba source, and TiO_2_∙4H_2_O (TH) for a Ti source, three different mixture samples, BH1TH (BH1 + TH), BH0TH (BH0 + TH), and BCTH (BC + TH), were heat-treated in the temperature range of 100–900 °C. BaTiO_3_ powders were synthesized at temperatures as low as 100 °C when sample BH1TH was subjected to MWH. Based on the growth exponent (*n*), the synthesis reactions were inferred to be diffusion-controlled processes (3 ≤ *n* ≤ 4) for MWH and interface-controlled processes (2 ≤ *n* ≤ 3) for CH. Current densities of approximately 0.073 and 0.022 mA/m^2^ were measured for samples BH1TH and BH0TH, respectively, indicating the generation of charged radicals by the interaction between the precursors and injected microwaves. The radicals were determined as OH^−^ groups by X-ray photoelectron spectroscopy and Fourier transform infrared spectroscopy.

## 1. Introduction

Microwaves are regarded as potential candidates for advanced technology sources in forthcoming industrial engineering applications, such as communications, materials science, and biomedical applications [1,2,3]. In particular, research groups have reported that microwave-assisted heating (MWH) in powder synthesis and sintering processes demonstrate high applicability, high energy efficiency, a low reaction temperature, and a short reaction time [4,5,6]. Thirumalai et al. [4] confirmed that BaTiO_3_ powders were synthesized at 1000 °C using the MWH method, which resulted in a shorter processing time of approximately 450 min compared to 520 min of conventional heating (CH). Guo et al. [5] reported that the application of microwaves in the hydrothermal method for the synthesis of BaTiO_3_ powders showed various advantages, such as time-saving advantages and a low reaction temperature when compared to a typical hydrothermal method. Thridandapani et al. [6] demonstrated that 8 mol% yttria-doped ZrO_2_ ceramics could be sintered with an activation energy of approximately 200 kJ/mol when produced by MWH, which was much lower than that (~520 kJ/mol) prepared by CH.

It is well known that dielectric materials are highly reactive to microwaves, which is described as dielectric heating caused by the reorientation of electric dipoles in response to the electric field of the microwaves [7]. The microwave power (P) absorbed in the materials is generally expressed by Equation (1) [8] as follows:

(1) $$P=\left(\omega \varepsilon_{0}\varepsilon^{\prime }tan\delta \right){\left|E\right|}^{2},$$

where ω is the frequency of the applied microwaves, $\varepsilon_{0}$ is the dielectric constant of the free space, $\varepsilon^{\prime }$ and tanδ are the real parts of the dielectric constant and the loss tangent of the materials, respectively, and *E* is the applied electric field strength. Based on this equation, MWH is significantly affected by the dielectric properties of the materials. Several researchers have investigated the enhancement in microwave effects on powder synthesis and sintering using various ionic materials with high dielectric constants and losses [9,10,11,12,13]. In contrast, it has been reported that hydroxyl and water molecules have high dielectric constants and loss tangents at microwave frequencies [14]. Therefore, precursors having hydroxyl and water molecules are potential candidates for powder synthesis via MWH. Our previous preliminary research on the application of microwave irradiation in the synthesis of BaTiO_3_ powders [15] confirmed that initial sources having hydroxyl and water molecules had a lower synthesis temperature compared to those without polar radicals.

We investigated the effect of the precursor polar radicals on the crystallization kinetics of BaTiO_3_ powders prepared via MWH. Here, we report how the precursors having hydroxyl and water molecules contributed to the crystallization of BaTiO_3_ powders during MWH. The values of the crystallization exponent and activation energy for the formation of the BaTiO_3_ phase were estimated in terms of the precursor types. In addition, the charge fluxes were measured under a DC-type electrical bias during the MWH process, and the charged radicals were identified by analyzing the chemical features and electronic structures of the constituents before and after the real-time charge flux measurements.

## 2. Experimental Details

### 2.1. Synthesis of BaTiO_3_ Powders

Three different types of Ba precursors and one type of Ti precursor were used to examine how the precursor type affects the crystallization of BaTiO_3_ powders prepared via MWH. The precursors for Ba were Ba(OH)_2_ (BH0; Alfa Aesar, Ward Hill, MA, USA, purity 94–98%), Ba(OH)_2_∙H_2_O (BH1; Alfa Aesar, Ward Hill, MA, USA, purity 95%), and BaCO_3_ (BC; Alfa Aesar, Ward Hill, MA, USA, purity 99.95%), and the precursor for Ti was TiO_2_∙4H_2_O (TH), which was an amorphous titania prepared by adding NH_4_OH solution (Samchun Chemicals, Pyeongtaek, Korea, concentration: 25.0–30.0%) to an aqueous TiOCl_2_ solution. The samples were prepared by mixing the precursors at a mole ratio of 1:1 and were denoted as BH0TH (BH0 + TH), BH1TH (BH1 + TH), and BCTH (BC + TH), respectively. For the synthesis of BaTiO_3_ powders, the samples were heated at a reaction temperature ranging from 100 to 900 °C for a soaking time of 0–60 min using the MWH and CH methods, respectively. The heating speeds for MWH and CH were 50 and 5 °C/min, respectively, and the furnaces were cooled naturally. The processing temperatures of the MWH and CH methods were measured using thermocouples placed in the hot zones of the microwave and conventional furnaces, respectively. MWH was conducted at a frequency of 2.45 GHz and a power of 2 kW using a microwave heating furnace (Unicera Co., UMF-04, Pyeongtaek, Korea) with a mono-mode reactor. CH was carried out at a power of 4 kW and a voltage of 220 V using an electrical furnace. 

### 2.2. Analysis of the Crystal Phase and Crystallization Activation Energy 

X-ray diffraction (XRD; PANalytical, Pro MRD, Malvern, UK) was utilized to examine the structural features of the synthesized powders; the Cu Kα line was used as the X-ray source, and the scan speed and step size were 2°/min and 0.01°, respectively. The crystallite size (*D*) of the BaTiO_3_ phase was calculated using the Scherrer equation [16] as follows:

*D* = 0.9*λ*/*B*cos*θ*, (2)

where *λ* is the wavelength of the X-ray (Cu Kα, 1.5406 Å), B is the full width at half maximum (FWHM), and *θ* is the center angle of the diffraction peak. The crystallization activation energy was estimated in terms of the source materials and synthesis methods to investigate the crystallization kinetics in the synthesis of BaTiO_3_ powders. The variation in the size of the crystallites synthesized at 100–400 °C for the MWH method and 600–900 °C for the CH method during the reaction time range of 0^+^ to 60 min was measured to calculate the activation energy for the BaTiO_3_ phase formation. 

### 2.3. Analysis of Charged Radicals in MWH

**Figure 1:**
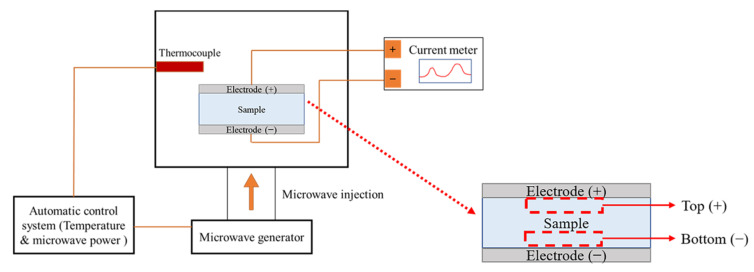
Schematic of real-time charge flux measurement system. A DC-type external bias was applied between the positive and negative electrodes of the specimens during microwave-assisted heating (MWH). Top (+) and bottom (−) regions of the specimen were designated as being near the positive and negative electrodes, respectively.

The presence of mobile charged radicals was examined by real-time measurements of the current for the different precursor types, as shown in Figure 1, using a current meter (Keithley, 2635A, Solon, OH, USA) with an applied DC-type external bias of 5 V during MWH. A cylindrical sample with a thickness of 6 mm and a radius of 5 mm was prepared by uniaxial pressing at a pressure of 1600 kg/cm^2^ for 2 min, and the top and bottom electrodes were prepared with Pt by sputtering. X-ray photoelectron spectroscopy (XPS; Thermo Fisher Scientific, K-Alpha, Waltham, MA, USA) with a monochromated Al Kα X-ray source was used to identify the charged radicals. The chemical states of the powders obtained from the top (+) and bottom (−) regions were examined after real-time measurements were carried out at an energy resolution of 0.1 eV, and the top (+) and bottom (−) regions were denoted as the regions near the positive and negative electrodes, respectively. In particular, the XPS spectra of Ti and O were deconvoluted based on their reference electronic energy states. Fourier transform infrared (FT-IR, Bruker VERTEX 80V, Billerica, MA, USA) was used to examine the variation in the chemical features of samples BH1TH and BH0TH under the heating condition of 100 °C for soaking times of 10 and 60 min, respectively. The scan number and resolution of the FT-IR measurements were determined to be 32 and 4 cm^−1^, respectively.

## 3. Results and Discussion

### 3.1. Structural Features of Synthesized Powders

**Figure 2:**
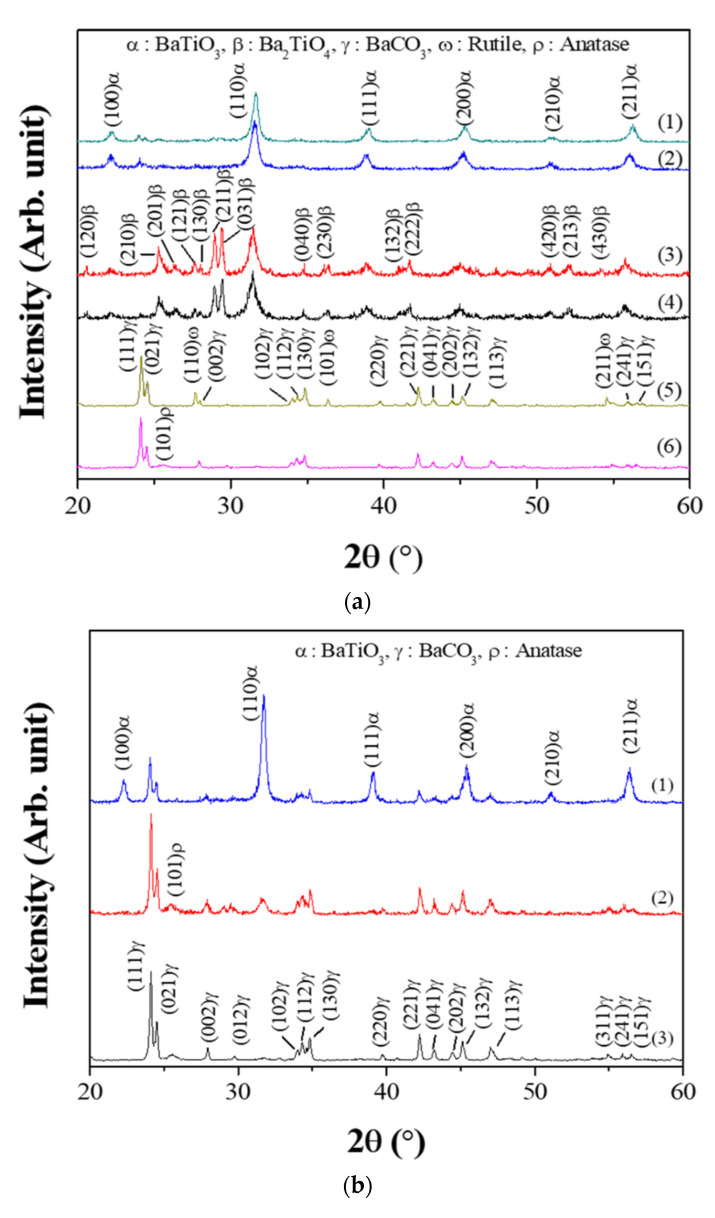
X-ray diffraction (XRD) spectra of synthesized powders. For MWH of duration 60 min, (**a**) the powders were synthesized at (1) 300 and (2) 100 °C using sample BH1TH, at (3) 300 and (4) 100 °C using sample BH0TH, and at (5) 300 and (6) 100 °C using sample BCTH, respectively. For conventional heating (CH) duration of 60 min, (**b**) the powders were synthesized at (1) 800, (2) 600, and (3) 400 °C using sample BH1TH, respectively.

Figure 2 shows the XRD results of the powders prepared from samples BH1TH, BH0TH, and BCTH using the MWH and CH methods. For the powders prepared by MWH, as shown in Figure 2a, those using sample BH1TH had a dominant BaTiO_3_ (JCPDS #75-0212) structure and a small amount of the BaCO_3_ (JCPDS #05-0378) phase at the reaction temperatures of both 100 and 300 °C. There was little variation in the peak intensity of the BaTiO_3_ and BaCO_3_ phases with an increase in the reaction temperature from 100 to 300 °C. On the other hand, the powders synthesized from sample BH0TH exhibited both the BaTiO_3_ and Ba_2_TiO_4_ (JCPDS #38-1481) phases at 100 and 300 °C; the main peaks with a similar intensity ratio were obtained at 31.3° and 29.4° for BaTiO_3_ and Ba_2_TiO_4_, respectively. The peak intensity of the Ba_2_TiO_4_ phase increased slightly with an increase in the reaction temperature from 100 to 300 °C. In contrast, the powders synthesized from sample BCTH showed a mixture of BaCO_3_ and anatase-like TiO_2_ (JCPDS #12-1272) phases at 100 °C. There was no reaction between the precursors for Ba and Ti at 100 and 300 °C. As the reaction temperature increased to 300 °C, a phase transition from anatase to rutile (JCPDS #75-1537) took place in the TiO_2_ structure. In the case of the powders prepared from sample BH1TH by the CH method, as shown in Figure 2b, those synthesized at 400 °C exhibited a dominant BaCO_3_ phase with a small fraction of an anatase-like TiO_2_ phase. When the reaction temperature was increased to 600 °C, the BaCO_3_ and TiO_2_ phases remained dominant and a small fraction of the BaTiO_3_ phase was apparently produced. At 800 °C, the BaTiO_3_ phase was dominant with a small amount of the BaCO_3_ phase.

In the present study, BaTiO_3_ powders were well synthesized at temperatures as low as 100 °C when samples with hydroxyl and water molecules were treated by MWH. It is known that precursors containing water molecules have a high reactivity owing to their high surface energy [17]. In addition, hydroxyl and water molecules have a naturally strong electrical polarity and could be highly responsive to the electromagnetic fields of microwaves due to their dipoles. Therefore, BH1TH and BH0TH were expected to efficiently absorb microwave-related energy to reach a high-energy state, so that the crystallization of the BaTiO_3_ phase could be achieved even at a low temperature of 100 °C. In contrast, in BCTH, crystallization and phase transition occurred only for TH. This result indicated that the synthesis temperature of 100 °C for BCTH via MWH was insufficient to overcome the energy barrier for the reaction between the Ba and Ti precursors. In the case of BH1TH treated by the CH method, the crystallization of source TH to the anatase phase and the phase transition of source BH1 to the BaCO_3_ phase occurred without any reaction between the precursors to form the BaTiO_3_ phase below 600 °C; the reaction between sources BH1 and TH occurred at temperatures higher than 600 °C. The reaction temperature required for the synthesis of the BaTiO_3_ phase by a conventional solid-state reaction is known to be over 600 °C when the BaCO_3_ and TiO_2_ phases are used as the precursors for Ba and Ti ions [18,19]. There was little noticeable enhancement of the reaction due to the high surface energy of the precursors in the CH method. 

### 3.2. Activation Energy Estimation for BaTiO_3_ Phase Formation

The crystallization kinetics was estimated to examine the effect of MWH on the formation of the BaTiO_3_ phase in terms of the precursor types. Based on the crystallite size obtained from the XRD results, the crystallization activation energy of the BaTiO_3_ phase was calculated as follows [20]:

(3) $$G^{n}-G_{0}^{n}=k\left(t-t_{0}\right),$$

where *G* and *G*_0_ are the crystallite size of the BaTiO_3_ structure for a specific reaction time (*t*) and the reaction time of 0^+^ (*t*_0_), respectively, *n* is the growth exponent related to the crystallization mechanism, and *k* is the rate constant for the reaction. The best-fit method was used to evaluate the exponent (*n*) based on Equation (3); the value of *n* was determined under the condition of highest reliability [21,22]. The respective reliability was estimated by the least squares method when a linear fitting was performed; the variation in the crystallite size (*G^n^*) with time (**t**) was measured at a specific temperature for a particular value of *n* in Equation (3).

**Figure 3:**
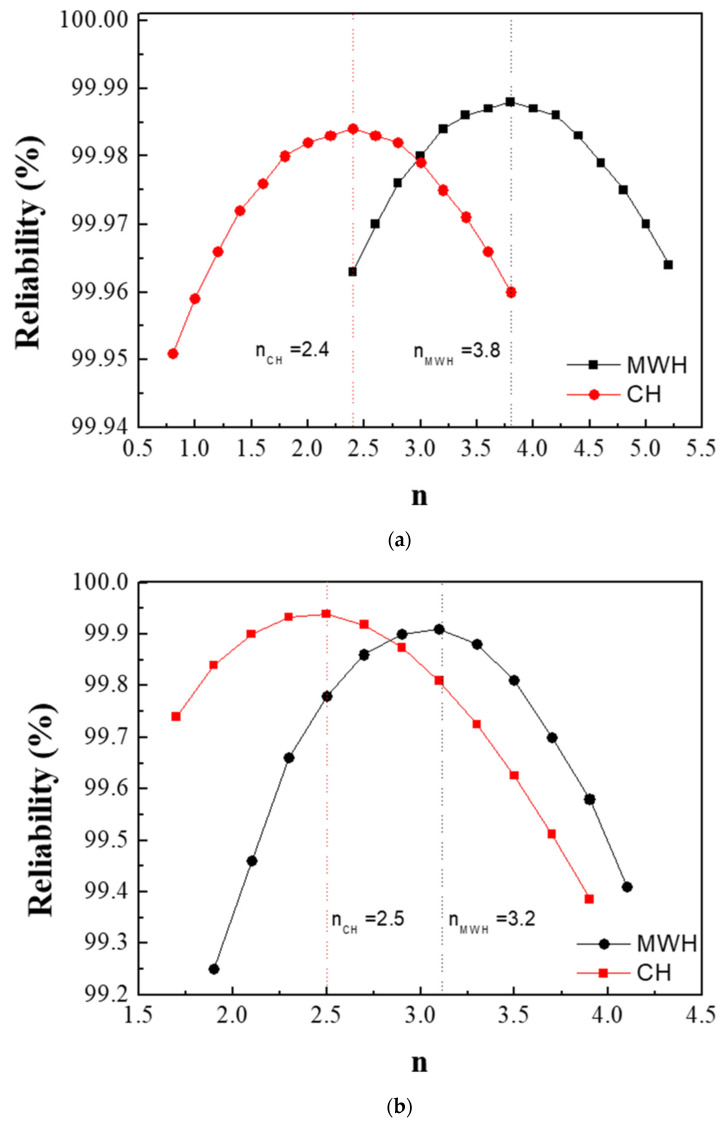

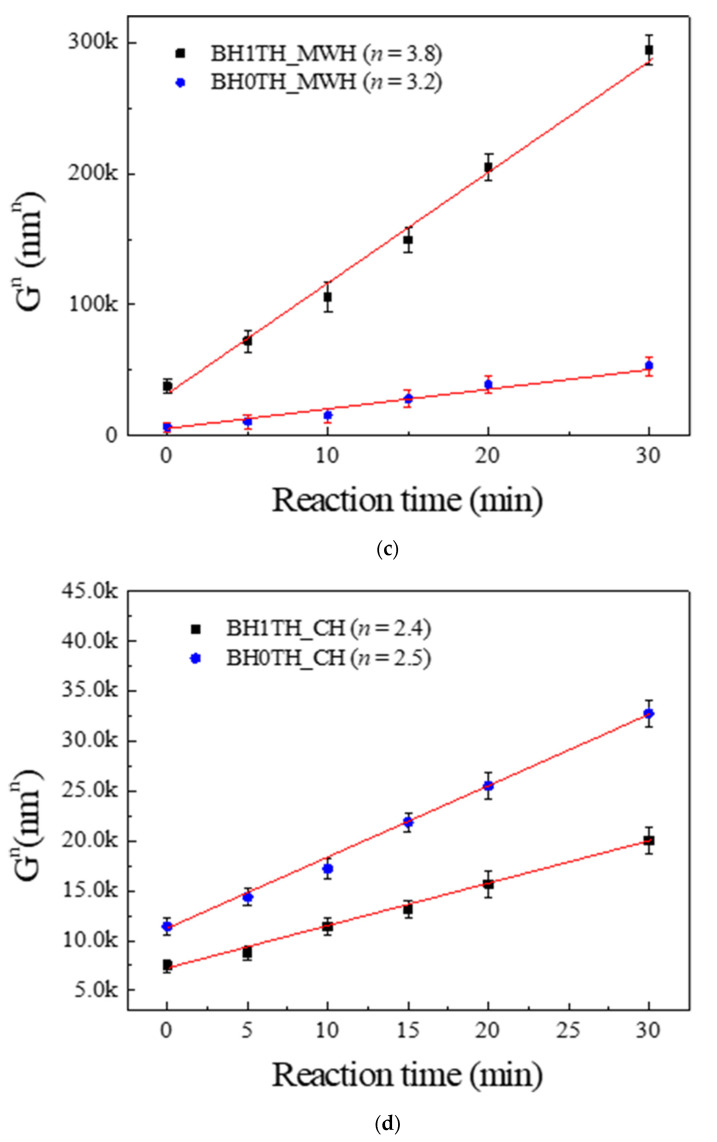

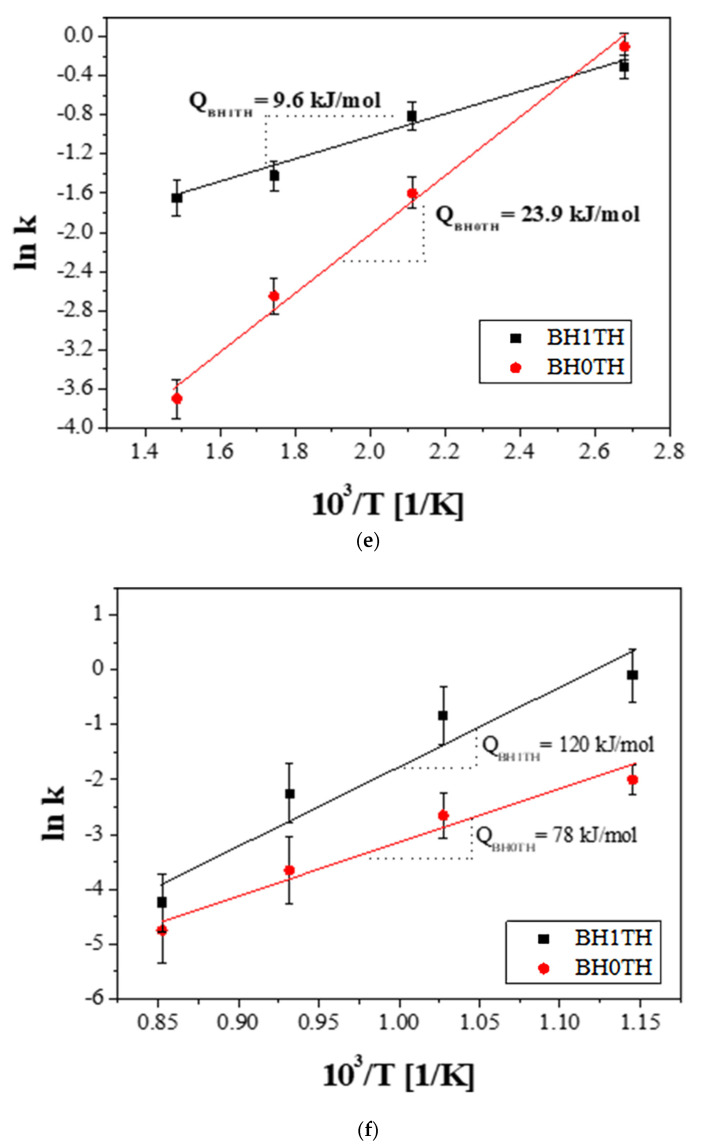
Growth exponents (*n*) in the synthesis of BaTiO_3_ powders from samples (**a**) BH1TH and (**b**) BH0TH; the values of *n* were determined under the best-fit conditions. Variation in crystallite size (*G^n^*) of BaTiO_3_ powders with time for specific values of *n*; the powders were prepared at 100 °C via (**c**) MWH and at 800 °C via (**d**) CH. Crystallization activation energies (*Q*) were estimated based on the relationship between ln k and 10^3^/T. The values of *Q* for the formation of BaTiO_3_ powders prepared by (**e**) MWH and (**f**) CH were calculated using the slope of the relationship, respectively.

Figure 3a,b show the values of n obtained from the powders prepared from samples BH1TH and BH0TH in terms of the heating methods. The values of *n* were determined to be 3.8 and 2.4 when sample BH1TH was treated by MWH and CH, respectively. On the other hand, in the synthesis carried out for sample BH0TH by MWH and CH, the values of *n* were 3.2 and 2.5, respectively. Figure 3c,d show two sets of experimental results showing linear fitting at the highest reliability; the reliabilities of the *n* values of sample BH1TH were obtained to be 99.988% for MWH and 99.984% for CH, whereas those of sample BH0TH were 99.910% for MWH and 99.939% for CH. The fitting lines drawn for specific values of *n* were well matched with the experimental data points of the variation in crystallite size (*G^n^*) with time. 

The preparation of powders by MWH had *n* values (3.8, 3.2) higher than those (2.4 and 2.5) for the synthesis of powders by CH. It is well known that a conventional solid-state reaction, in which interface-controlled crystal growth is known to be the dominant mechanism, exhibits an *n* value of ~2 [23,24]. On the other hand, if the *n* value ranges between 3 and 4, diffusion-controlled crystal growth is considered dominant for the reaction. This value can vary with the reactant energy states, processing techniques, and impurities in the precursors [25]. In the case of the MWH method in the present study, *n* values ranging from 3 to 4 indicate that the diffusion-controlled growth mechanism is dominant for the reactions. In contrast, the synthesis of powders prepared from samples BH1TH and BH0TH by the CH method showed *n* values of ~2.5. These results indicate that crystallization by the CH method is mainly dependent on interface-controlled crystal growth. 

The crystallization activation energy can be calculated using the Arrhenius equation as follows:

ln *k* = ln *k*_0_ − *Q*/*R*T, (4)

where *k*_0_ is the pre-exponential constant, *Q* is the activation energy, *R* is the gas constant, and T is the reaction temperature (K). The activation energies for the crystallization of the BaTiO_3_ phase prepared from BH1TH and BH0TH were calculated from the slope of the plots of ln *k* versus 10^3^/T in terms of the heating method. When the powders were prepared by MWH at 100–400 °C, as shown in Figure 3e, the activation energies for BH1TH and BH0TH were determined to be approximately 9.6 and 23.9 kJ/mol, respectively. In contrast, when the powders were prepared by CH at 600–900 °C, as shown in Figure 3f, the activation energies for samples BH1TH and BH0TH were calculated to be 120 and 78 kJ/mol, respectively.

Based on the difference in the values of *n* and the activation energy, it is evident that the crystallization mechanism for BH1TH and BH0TH was critically dependent on the MWH and CH methods. Therefore, real-time charge flux measurements under a DC-type electrical bias were carried out to determine the detailed features of the crystallization kinetics in MWH.

### 3.3. Real-Time Measurements of Charge Fluxes during MWH

**Figure 4:**
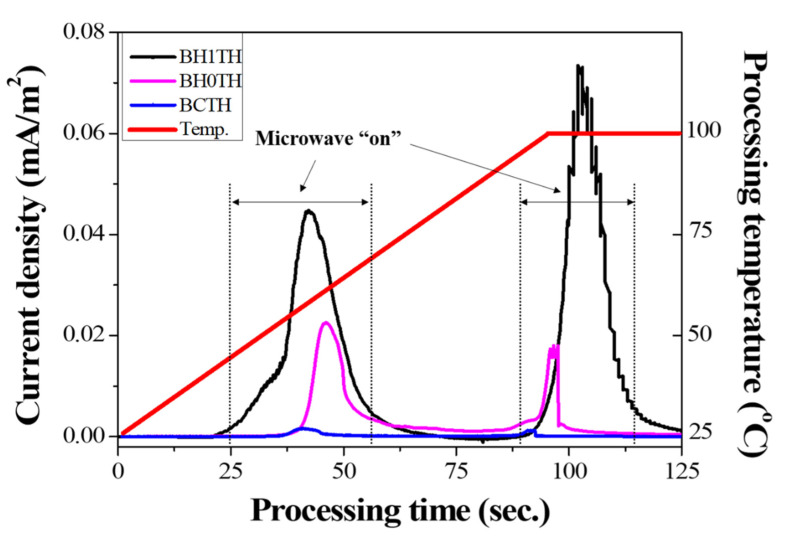
Results of real-time current density measurements for three different sample types. The current densities were obtained under a DC-type external bias applied between the top and bottom electrodes of the specimens during MWH.

In Figure 4, the variation in the current density was measured under a DC-type external bias during MWH for each sample; the process was carried out in the temperature range of room temperature to 100 °C at a heating rate of 50 °C/min. The microwave power was automatically turned on for processing time ranges of 25–65 and 80–110 s to adjust the processing temperature, and a DC-type electrical bias of 5 V was applied to the samples in the MWH chamber.

In the case of sample BH1TH, the current densities measured 0.045 and 0.073 mA/m^2^ when the microwaves were switched on in the processing time ranges of 25–65 and 80–110 s, respectively. Sample BH0TH showed current densities of 0.022 and 0.015 mA/m^2^ when the microwaves were turned on in the time ranges of 40–55 and 80–95 s, respectively. When the microwaves were turned off, the current densities of both samples BH1TH and BH0TH decreased to zero. In contrast, sample BCTH showed a current density of <0.001 mA/m^2^ when the microwaves were turned on, which is a negligible value compared to those of the other samples.

It was found that noticeable current densities were measured only when the microwaves were turned on, indicating that the charged radicals evolved because the sources responded to the injected microwaves. In addition, the value of the current density was dependent on the type of precursor in the order of samples BCTH < BH0TH < BH1TH. Freeman et al. [26] confirmed that an ionic current was generated by the transport of mobile ions in halide salt crystals when the crystals were exposed to microwaves. Their study revealed that the magnitude of the ionic current was proportional to the microwave power. Booske et al. [27] reported that the ionic current observed under electromagnetic fields was caused by a ponderomotive force. This could enhance mass transport and, as a result, enhance the solid-state reaction rates during MWH of ceramic materials. In our experiments, the generation of charged radicals in samples BH1TH and BH0TH appeared to enhance the energy state of the precursors for Ba and Ti and, as a result, the presence of charged radicals could affect the crystallization kinetics, particularly the reaction rates, and the temperature for the synthesis of the BaTiO_3_ phase. 

### 3.4. Chemical Features of Samples after Heat Treatment

**Figure 5:**
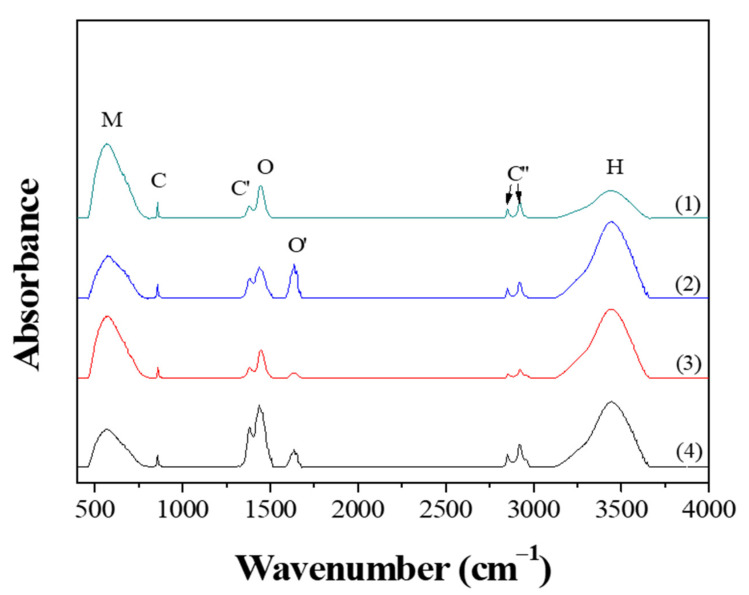
FT-IR results of the powders prepared by MWH at 100 ℃ for (1,3) 60 and (2,4) 10 min using samples BH1TH (1,2) and BH0TH (3,4).

Figure 5 shows the FT-IR spectra obtained from the powders prepared from samples BH1TH and BH0TH by MWH at 100 °C for 10 and 60 min, respectively. When the powders were synthesized for 10 min, both BH1TH and BH0TH showed characteristic bands in the ranges of 450–800, 850, 1370, 1400–1520, 1580–1700, 2800–3000, and 3100–3700 cm^−1^, which are denoted as M, C, C′, O, O′, C′′, and H, respectively. Band M is assigned to the TiO_6_ octahedra in the BaTiO_3_ phase [28]. Peaks C and C′ are associated with the asymmetric deformation and stretching vibrations of the BaCO_3_ phase, respectively [29]. Band O is the sum of several overlapping peaks in the region; these are related to a characteristic bond of the BaTiO_3_ phase and the OH bonds for Ba(OH)_2_ as well as hydrates [30,31]. Band O′ is associated with the lattice vibrations of the BaO phase [31]. The multiple peaks of C′′ are caused by the C–H stretching vibrations of hydrogen carbonate, which normally exist in C-containing impurity species such as BaCO_3_ [32]. Band H is generated by the surface-absorbed hydroxyl (OH_Surf._) groups [33]. Powders prepared from sample BH1TH heated for 60 min showed that band O′ disappeared completely and the intensity of peaks M and O increased significantly, whereas the intensity of peak H decreased; as the heating time was increased from 10 to 60 min, the intensity of band H for sample BH1TH was halved. On the other hand, compared to the variation in the intensity of peaks for sample BH1TH with the heating time, there was less variation in the peak intensity of sample BH0TH with the increase in the reaction time from 10 to 60 min. The intensity of peak M increased slightly and those of peaks O and O′ decreased noticeably.

When BH1TH and BH0TH were heated at 100 °C for 60 min, the fraction of TiO_6_ octahedra for the BaTiO_3_ phase increased whereas that of OH_Surf._ decreased, indicating that the crystallinity of the BaTiO_3_ phase increased with the decreasing OH_Surf._ fraction. It has also been reported that the fraction of OH_Surf._ on oxide materials decreased as the materials were treated by CH at temperatures over 400 °C [34]. Therefore, it is believed that a decrease in OH_Surf._ after MWH at 100 °C for 60 min was due to the high reactivity of OH_Surf._ to the injected microwaves, which appears to be associated with the crystallization kinetics of the BaTiO_3_ phase formation during the MWH process. 

### 3.5. Determination of Mobile Charged Radicals in MWH

**Figure 6:**
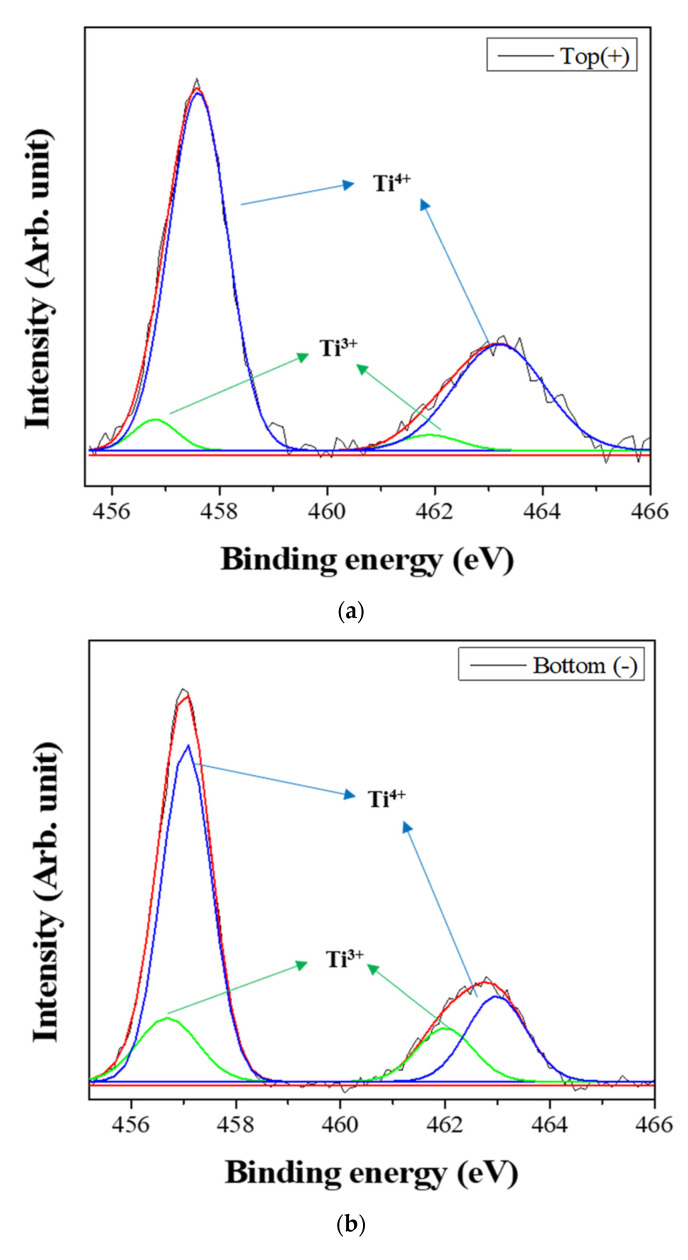

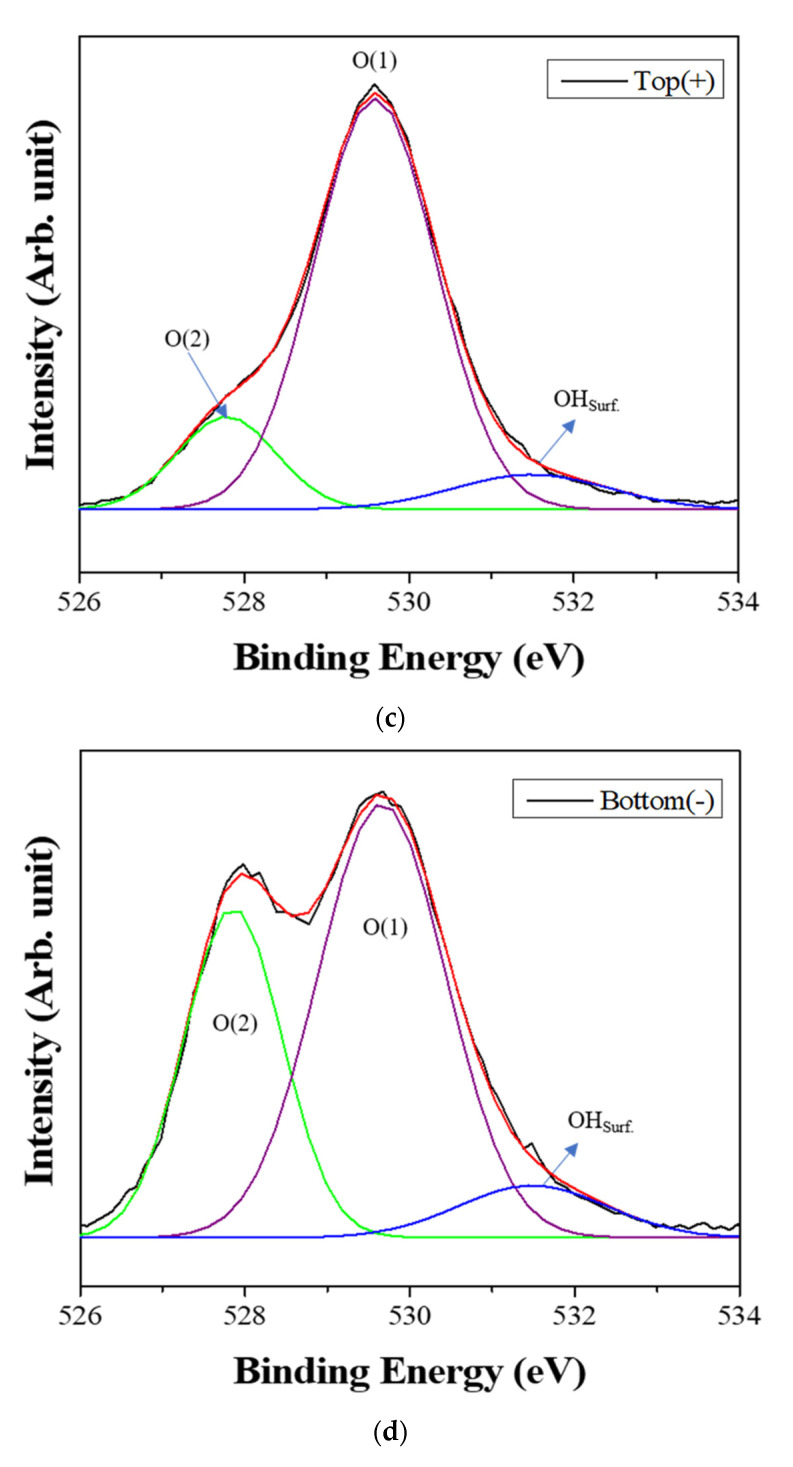

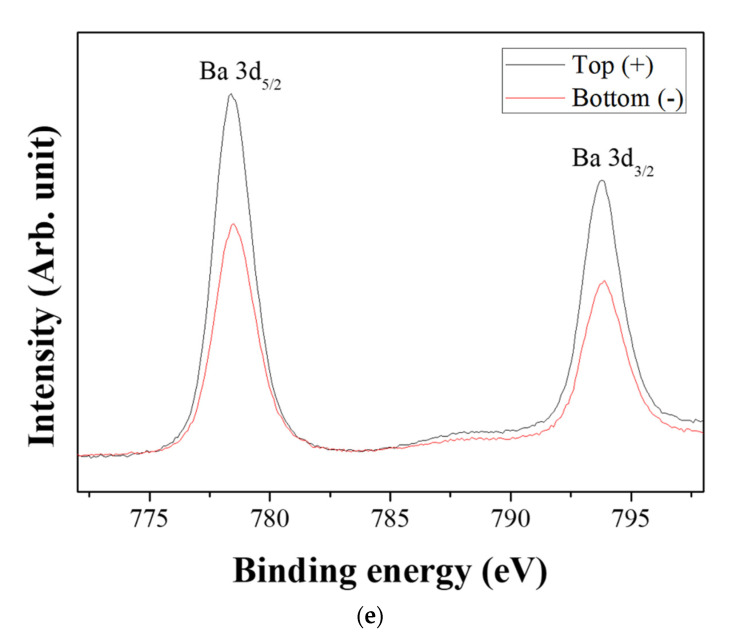
X-ray photoelectron spectroscopy (XPS) spectra of the powders prepared from sample BH1TH obtained after the real-time charge flux measurements during MWH. The spectra were recorded from the (**a**,**c**) top and (**b**,**d**) bottom regions for Ti (**a**,**b**) and O (**c**,**d**); (**e**) XPS spectra of Ba 3d_5/2_ and 3d_3/2_ were measured from both the top and bottom regions. Deconvolution was carried out based on the chemical states of Ti^3+^ and Ti^4+^ for the Ti 2p edge, and O(1), O(2), and OH_Surf._ for the O 1s edge.

Figure 6 shows the XPS spectra of the powders prepared from sample BH1TH, which were obtained from the top (+) and bottom (−) regions, as shown in Figure 1, after real-time charge flux measurements. Figure 6a,b show the deconvoluted XPS results of the Ti 2p edge of the powders obtained from the top (+) and bottom (−) regions; the spectra of the Ti 2p edge were deconvoluted into two components: Ti^3+^ states (near 456.8 and 462.0 eV) and Ti^4+^ states (near 457.9 and 463.6 eV) [35]. Based on the intensity of the respective spectra, the fraction of Ti^3+^ states for samples obtained from the top (+) and bottom (−) regions were determined to be 7.2% and 25.5%, respectively. Figure 6c,d show the XPS peaks of the O 1s edge for the samples obtained from the top (+) and bottom (−) regions, respectively. The spectra were deconvoluted based on three electronic energy states at 529.4 eV (O(1)), 527.8 eV (O(2)), and 531.2 eV (OH_Surf._) [35,36]; state O(1) is assigned to the oxygen atoms in a normal BaTiO_3_ lattice, while O(2) is associated with oxygen atoms in the presence of oxygen vacancies in their neighbors [35]. The spectrum obtained from the bottom (−) region has a fraction of 33.8% for state O(2), which is noticeably higher than that (12.1%) of the top (+) region. However, there was little variation in the fraction of OH_Surf_. with the electrode. In Figure 6e, two symmetric peaks of the Ba 3d_5/2_ edge at 778.5 eV and the 3d_3/2_ edge at 793.8 eV were observed, and there was little difference in the shape of the peaks for the Ba 3d edge between the top (+) and bottom (−) regions.

Based on the spectral intensity, it was found that the fractions (25.5% and 33.8%) of the Ti^3+^ and O(2) states in the top (+) region were higher than those (7.2% and 12.1%) in the bottom (−) region. It is evident that the negatively charged oxygen-related radicals are generated by the interaction between the precursors and the injected microwaves and subsequently migrate from the negative to the positive electrode under a DC-type external bias. In addition, the FT-IR results showed that the hydroxyl groups of the sources reacted strongly to the injected microwaves at 100 °C. It is highly likely that the oxygen-related radicals are hydroxyl groups, which were generated by the interaction between the precursors and the injected microwaves. It has been reported that two types of OH_Surf_ groups could occur on the surface of metallic oxides (M=O, such as TiO_2_); these include the terminal OH and the bridge OH based on the coordination states [37]. Generally, the bonding strength of the bridge OH is higher than that of the terminal OH. Therefore, it seems that the OH^−^ radicals could initially be generated from the terminal OH by the application of microwaves, which would enhance the reaction between the precursors for Ba and Ti ions in electromagnetic fields. When the microwave power is sufficiently high to generate the two different OH groups, the negatively charged OH^−^ radicals migrate from the negative to the positive electrode by the application of the DC-type external bias, causing a variation in the concentration of oxygen vacancies (O(2)) as well as that of Ti^3+^ with the distance from positive to negative electrodes in the specimen after the real-time charge flux measurements. 

From the *n* values, it is evident that the crystallization mechanism of BH1TH is diffusion-controlled for MWH and interface-controlled for CH. Based on the FT-IR results (Figure 5), the formation of charged radicals becomes clear under the microwaves, and the measured charged particles such as OH^−^ are expected to easily respond to the oscillation of the electrical fields of the injected microwaves, resulting in highly energetic states (OH^−^)*. In addition, the crystallization activation energy for BH1TH via MWH turned out to be 9.6 kJ/mol, which is approximately 1/12 of that measured for BH1TH via CH. As a result, it is highly likely that the reactants of the precursors for Ba and Ti, such as (BaO)* and (TiO_2_)*, are in their high-energy states, so that the energy barrier to be surmounted by the reactants becomes smaller. A diffusion-controlled mechanism indicates that molecules and radicals such as (BaO)*, (TiO_2_)*, and (OH^−^)* are produced from the precursors during MWH, and the formation of the BaTiO_3_ phase occurs at temperatures as low as 100 °C. The formation rate is predominantly controlled by the diffusion-related transport of (BaO)* to (TiO_2_)* molecules in electromagnetic fields. In contrast, an interface-controlled mechanism was applied to the formation of BaTiO_3_ powders from samples BH1TH and BH0TH via CH. In this process, the BaTiO_3_ phase is synthesized at the interface between the reactants for Ba and Ti, such as BaO and TiO_2_, at temperatures over 600 °C. The growth rate is predominantly affected by the crystallization rate at the interface, which requires a larger activation energy as well as higher temperatures than those measured for BH1TH via MWH.

## 4. Conclusions

Nanocrystalline BaTiO_3_ powders were synthesized at 100 °C by subjecting samples containing hydroxyl and water molecules to MWH. Based on the n values, it was inferred that the synthesis process via MWH with samples having polar molecules is a diffusion-controlled mechanism (3 ≤ *n* ≤ 4); an interface-controlled mechanism (2 ≤ *n* ≤ 3) is applied to the synthesis process via CH. The activation energy for the formation of the BaTiO_3_ phase prepared via MWH was estimated to be 9.6 kJ/mol for sample BH1TH, which was lower than that (23.9 kJ/mol) for sample BH0TH, and both were much lower than that for samples BH1TH (120 kJ/mol) and BH0TH (78 kJ/mol) prepared by the CH method. In addition, when the microwaves were turned on, current densities of up to approximately 0.073 and 0.022 mA/m^2^ were measured for the BH1TH and BH0TH samples, respectively, indicating the generation of charged radicals during the MWH process. Based on the XPS results, it was determined that the radicals were negatively charged oxygen-related radicals. The FT-IR results showed that the hydroxyl groups of the sources reacted strongly to the injected microwaves during MWH. As a result, the charged radicals were determined to be OH^−^ groups, and these were generated by the interaction between the precursors and the injected microwaves, which enhanced the kinetic conditions for the crystallization of BaTiO_3_ phases in electromagnetic fields. 

## Data Availability

The data presented in this study are available on request from the corresponding author.
